# Fracture morphology–driven surgical strategy in ankylosing spondylitis: when does sagittal imbalance mandate pedicle subtraction osteotomy?

**DOI:** 10.1186/s12891-026-09806-w

**Published:** 2026-04-11

**Authors:** In-Seok Son, Yong-Chan Kim, Sung-Min Kim, Xiongjie Li, Young-Jik Lee, Maolin Jin, Billy Francis Hung

**Affiliations:** Department of Orthopaedic Surgery, Kyung Hee University Hospital at Kang Dong, College of Medicine, Kyung Hee University, 892 Dongnam-ro, Gangdong-gu, Seoul, 134-727 South Korea

**Keywords:** Ankylosing spondylitis, Thoracolumbar fracture, Fracture morphology, Surgical strategy, Sagittal imbalance, Pedicle subtraction osteotomy, Predictive modeling, Decision tree

## Abstract

**Background:**

Thoracolumbar fractures in patients with ankylosing spondylitis (AS) pose unique challenges due to altered spinal biomechanics and sagittal imbalance. Optimal surgical strategies remain controversial, and objective criteria for selecting between deformity-corrective and fusion-only procedures are lacking.

**Methods:**

We retrospectively reviewed 41 AS patients with thoracolumbar fractures who underwent surgical treatment. Patients were categorized as vertebral body (VB) type or intervertebral space (IS) type fractures. The primary outcome was surgical strategy selection, defined as pedicle subtraction osteotomy (PSO) versus non-PSO procedures. Propensity score matching (PSM) was performed using preoperative clinical and radiographic parameters to minimize selection bias in comparing outcomes between fracture types. Radiographic outcomes, pain improvement, and complication rates were compared. Predictive modeling using logistic regression and decision tree analysis was conducted to identify determinants of surgical strategy.

**Results:**

A total of 41 patients (24 VB-type, 17 IS-type) were included. The VB group exhibited significantly greater preoperative sagittal imbalance and underwent PSO more frequently, while IS-type fractures were treated with posterior spinal fusion (PSF). PSM analysis (*n* = 17 per group) confirmed greater radiographic correction in the VB group without differences in pain outcomes. The decision tree model achieved an accuracy of 82.9% and AUC = 0.88 (95% CI: 0.85–0.99), with C7SVA > 222.6 mm as the primary decision criterion. Multivariable logistic regression identified C7SVA as the only statistically significant predictor (OR = 1.013; 95% CI: 1.001–1.026; *p* = 0.039). These findings suggest promising but preliminary discriminative ability, and external validation is warranted.

**Conclusions:**

In AS patients with VB-type thoracolumbar fractures, preoperative C7SVA > 222.6 mm represents a data-supported threshold for PSO, while C7SVA > 186.8 mm combined with BMD ≤ − 2.25 identifies a secondary risk group warranting PSO consideration. PSF alone appears sufficient for IS-type injuries with relatively preserved sagittal alignment. Preoperative sagittal profiling and bone mineral density assessment enable individualized, data-driven surgical planning in this high-risk population.

**Supplementary Information:**

The online version contains supplementary material available at 10.1186/s12891-026-09806-w.

## Introduction

Ankylosing spondylitis (AS) is a chronic inflammatory disease characterized by progressive spinal ankylosis and osteoporosis, resulting in a rigid and brittle spine [1, 2]. These structural changes predispose patients to unstable thoracolumbar fractures, often involving all three spinal columns, even after minor trauma [1, 3]. Depending on the anatomical location of injury, fractures may occur through the vertebral body (VB-type) or the intervertebral space (IS-type), each presenting distinct patterns of instability [4]. Due to altered biomechanics, these fractures frequently result in kyphotic deformity, delayed union, or neurological deficits [4, 5]. 

Surgical stabilization is commonly required to restore alignment and prevent neurological deterioration, especially in patients with progressive sagittal imbalance [6, 7]. However, the optimal surgical strategy remains controversial. Pedicle subtraction osteotomy (PSO) allows for sagittal correction but is invasive and carries higher complication risks [7, 8]. In contrast, posterior spinal fusion (PSF), with or without structural bone grafting, may suffice in less deformed spines but offers limited corrective capacity [9–11]. No standardized, objective criteria currently exist to guide the selection between these strategies in the acute or subacute fracture setting.

Fracture morphology plays a key role in surgical decision-making. As described by Caron et al., [12] VB-type fractures typically result in greater disruption of anterior and middle columns and more severe kyphotic collapse, often necessitating corrective procedures such as PSO. In contrast, IS-type injuries tend to preserve partial structural integrity and may be managed with fusion alone. However, the extent to which fracture morphology, together with preoperative sagittal alignment parameters, should drive the choice between PSO and non-PSO strategies has not been systematically evaluated. Identifying objective radiographically-based thresholds for this binary surgical decision represents an unmet clinical need.

This study aimed to evaluate the safety and outcomes of PSO-based deformity correction compared to non-PSO fusion strategies in AS patients with thoracolumbar fractures, with surgical strategy selection (PSO vs. non-PSO) as the primary outcome, and to identify preoperative radiographic thresholds that could objectively guide this surgical decision. We hypothesized that VB-type fractures, characterized by greater preoperative sagittal imbalance, would more frequently require PSO, and that specific radiographic parameters, particularly C7SVA, could serve as decision-guiding thresholds. Given the inherent differences in baseline sagittal alignment between fracture types as a potential source of selection bias, propensity score matching (PSM) was employed to enable a more balanced comparison of outcomes. Multivariable logistic regression and decision tree analysis were subsequently applied to identify the strongest preoperative predictors of surgical strategy selection.

Due to the rarity of ankylosing spondylitis (AS) and the even lower occurrence of thoracolumbar fractures within this patient population, a priori power analysis was not conducted. Given the challenges in reliably estimating both the incidence and effect sizes for such an uncommon condition, power calculation was deemed impractical in the context of this retrospective study. The reported global prevalence of AS ranges from 0.1% to 1.4%, and the incidence of spinal fractures among AS patients has been reported between 5% and 15%, depending on age and disease duration [1, 2]. 

## Methods

### Study design and patient selection

This was a retrospective cohort study conducted at a single tertiary referral center. We reviewed patients diagnosed with ankylosing spondylitis (AS) who underwent surgical treatment for thoracolumbar fractures between May 2013 and January 2019. Inclusion criteria were: (1) AS diagnosis confirmed by the modified New York criteria; (2) acute thoracolumbar fracture between T9 and L2 confirmed by radiography or CT; (3) surgical treatment with available pre- and postoperative radiographic data and clinical follow-up ≥ 24 months.

Exclusion criteria included: (1) multiple simultaneous spinal fractures; (2) active spinal infection; (3) malignancy; (4) history of spinal or hip surgery; (5) incomplete imaging or clinical data.

This study was approved by the institutional review board (IRB No. KHNMC 2025-05-024), and the requirement for informed consent was waived due to the retrospective design and use of anonymized data.

### Fracture classification

Fracture morphology was categorized using a simplified version of Caron et al.’s anatomical classification [12]. Patients were grouped into two categories:

1. Vertebral body (VB) type: fractures traversing the vertebral body, typically disrupting anterior and middle columns.
2. Intervertebral space (IS) type: fractures localized to the disc space with relative preservation of vertebral body structure.

This classification was applied independently by two fellowship-trained spine surgeons using preoperative CT scans. Disagreements were resolved by consensus.

### Radiographic evaluation

Radiographic parameters were assessed using standing lateral radiographs at three time points: preoperatively, 3 months postoperatively, and at final follow-up (> 24 months). The following sagittal alignment metrics were measured: C7 sagittal vertical axis (C7SVA), Lumbar lordosis (LL), Global kyphosis (GK), Thoracic kyphosis (TK), Thoracolumbar kyphosis (TLK), Pelvic parameters: pelvic incidence (PI), pelvic tilt (PT), sacral slope (SS). All measurements were performed by two blinded spine surgeons. Interobserver discrepancies > 5° or > 10 mm was resolved by joint review.

### Surgical techniques

Four posterior-only surgical techniques were employed depending on fracture morphology and sagittal alignment: (1) In-situ PSF, (2) PSF with structural bone graft (BG), (3) Posterior osteotomy with PSF, (4) PSO.

Instrumentation typically spanned three or more vertebrae above and below the fracture site. The decision regarding osteotomy versus in-situ fusion was based on surgeon judgment and preoperative alignment.

### Propensity score matching

To minimize selection bias in comparing VB and IS fracture types, 1:1 propensity score matching (PSM) was conducted. Matching was performed using Optimal 1:1 matching using the Hungarian algorithm on logit-transformed propensity scores. The propensity score was calculated using logistic regression incorporating the following baseline covariate: Age, Sex, Bone mineral density (BMD), Preoperative sagittal alignment parameters (C7SVA, LL, GK, TLK). Covariate balance after matching was assessed using standardized mean differences (SMD), with SMD < 0.1 considered indicative of adequate balance. Seventeen matched pairs were identified. Baseline characteristics before and after matching are presented in Table 1. In the matched cohort, radiographic outcomes, pain scores (VAS), functional disability (ODI), and complication rates were compared between groups.

**Table 1:** Baseline characteristics of all patients and propensity score–matched cohort

Variable	Before Propensity Score Matching				After Propensity Score Matching (*n* = 17 pairs)			
	IS-type (*n* = 17)	VB-type (*n* = 24)	*p*-value	SMD	IS-type (*n* = 17)	VB-type (*n* = 17)	*p*-value	SMD
Age (years)	54.4 ± 11.0	53.9 ± 11.8	0.884	0.047	54.4 ± 11.0	53.4 ± 12.3	0.793	0.091
Sex, Male	14/17 (82.4%)	20/24 (83.3%)	1.000	0.026	14/17 (82.4%)	13/17 (76.5%)	1.000	**0.146**
BMD (T-score)	-0.6 ± 1.7	-0.8 ± 1.8	0.750	**0.103**	-0.6 ± 1.7	-0.7 ± 2.1	0.906	0.041
Pre-op C7SVA (mm)	139.9 ± 65.6	201.1 ± 60.7	**< 0.001**	**0.97**	139.9 ± 65.6	173.6 ± 45.7	0.056	**0.680**
Pre-op LL (°)	18.3 ± 12.6	4.9 ± 29.3	**0.039**	**0.59**	10.7 ± 14.9	11.4 ± 25.5	0.926	0.032
Pre-op GK (°)	59.2 ± 21.0	51.7 ± 19.0	0.243	**0.373**	59.2 ± 21.0	59.6 ± 13.8	0.949	0.022
Pre-op TLK (°)	33.5 ± 19.3	28.2 ± 16.7	0.351	**0.295**	33.5 ± 19.3	28.8 ± 18.9	0.478	**0.246**
Follow-up (years)	6.4 ± 3.1	6.3 ± 2.7	0.932	0.027	6.4 ± 3.1	5.9 ± 2.9	0.612	**0.176**
PSO	1/17 (5.9%)	18/24 (75.0%)	**< 0.001**	**1.983**	1/17 (5.9%)	13/17 (76.5%)	**< 0.001**	**2.058**

Values are mean ± SD or n/N (%). SMD, standardized mean difference; C7SVA, C7 sagittal vertical axis; LL, lumbar lordosis; GK, global kyphosis; TLK, thoracolumbar kyphosis; BMD, bone mineral density; PSO, pedicle subtraction osteotomy

Bold SMD values indicate SMD > 0.10, suggesting residual imbalance after matching

† Propensity scores were estimated using logistic regression with covariates: age, sex, BMD, pre-operative C7SVA, LL, GK, and TLK. Optimal 1:1 matching was performed using the Hungarian algorithm (minimum-cost assignment) on logit-transformed propensity scores

‡ PSO rate reflects the surgical strategy outcome variable, not a matching covariate; between-group difference is expected

### Clinical outcomes and complications

Patient-reported outcomes were assessed using: Visual Analog Scale (VAS) for back pain, Oswestry Disability Index (ODI). Postoperative complications were reviewed and classified into dural tears, infections, pneumothorax, and neurologic deficits.

### Statistical Analysis

Continuous variables were compared using the Student’s t-test or Wilcoxon rank-sum test, as appropriate. Categorical variables were analyzed using the chi-square or Fisher’s exact test. Paired t-tests were used to assess within-group changes in the matched cohort. A two-sided p-value < 0.05 was considered statistically significant.

For predictive modeling, multivariable logistic regression was performed to identify preoperative determinants of surgical strategy, with surgical approach (PSO vs. non-PSO) defined as the dependent variable. Initial candidate independent variables included age, sex, C7SVA, LL, BMD, GK, and TLK. Variables were selected based on clinical relevance and univariable screening; the final model included preoperative C7SVA, LL, and BMD as independent variables. Regression coefficients, odds ratios (OR), 95% confidence intervals (CI), and p-values for each variable are reported in Table 5. Variance inflation factors (VIF) were calculated to assess multicollinearity among sagittal parameters.

Decision tree analysis was performed using the classification and regression tree (CART) algorithm. Gini impurity was used as the splitting criterion, and the maximum tree depth was restricted to 3 to limit overfitting. All other parameters followed default Scikit-learn settings. Given the limited sample size (*n* = 41), leave-one-out cross-validation (LOOCV) was applied to evaluate model generalizability. Model performance was evaluated using the area under the receiver operating characteristic curve (AUC) with 95% confidence interval estimated via bootstrapping (1,000 iterations).

All analyses were conducted using Python 3.10 and Scikit-learn (v1.3.0). The analytical code is available from the corresponding author upon reasonable request.

## Results

### Baseline Characteristics

A total of 41 patients with ankylosing spondylitis (AS) and thoracolumbar fractures were included: 24 with vertebral body (VB-type) fractures and 17 with intervertebral space (IS-type) fractures. Baseline demographic and radiographic characteristics are summarized in Table 1. The mean age was 54.1 ± 11.3 years, and 85% were male. Groups were comparable in age, sex, BMI, and BMD (all *p* > 0.05).

VB-type fractures were associated with significantly greater sagittal imbalance, with a higher mean C7SVA (201.1 ± 60.7 mm vs. 139.9 ± 65.6 mm, *p* < 0.001) and lower LL (4.9 ± 29.3° vs. 18.3 ± 12.6°, *p* = 0.039) compared to the IS group. PSO was performed in 75.0% of VB-type patients (18/24), whereas 82.4% of IS-type patients (14/17) were treated with PSF alone or PSF with structural bone grafting (*p* < 0.001).

### Radiographic and Clinical Outcomes (Unmatched Cohort)

At a mean follow-up of 26.8 ± 5.1 months, both groups demonstrated significant within-group improvements in radiographic alignment. However, the VB group achieved significantly greater correction in global sagittal alignment compared to the IS group (ΔC7SVA: 132.7 ± 48.9 mm (VB) vs. 72.5 ± 61.8 mm (IS); *p* < 0.01, ΔLL: 26.5 ± 21.6° (VB) vs. 13.1 ± 15.5° (IS); *p* = 0.047) (Table 2). Both groups showed significant within-group reductions in pain intensity from baseline (ΔVAS: − 4.1 ± 1.1 (VB) vs. − 3.6 ± 1.2 (IS); *p* < 0.05 vs. base line); however, the between-group difference in pain improvement did not reach statistical significance (*p* = 0.142). (Table 3) Complication rates were numerically higher in the VB group (37.5% vs. 11.8%, *p* = 0.085) (Table 4), Although this difference did not reach statistical significance, the limited sample size may have reduced statistical power, and a type II error cannot be excluded. When stratified by surgical method, the PSO subgroup had the highest absolute number of complications; however, no statistically significant difference was observed across surgical subgroups, again likely reflecting insufficient power within each subgroup.

**Table 2:** Comparison of Radiologic Parameters in the IS Group and VB Group

	IS Group (*n* = 17)	VB Group (*n* = 24)	*p*-value
C7SVA (mm)			
Preop	139.9 ± 65.6	201.1 ± 60.7	< 0.001*
3 mos postop	67.4 ± 39.2	68.4 ± 20.9	0.718
2 year follow-up	70.8 ± 29.3	76.3 ± 24.9	0.324
Postop change	-72.5 ± 61.8	-132.7 ± 48.9	< 0.001*
GK (°)			
Preop	55.0 ± 23.5	51.6 ± 19.8	0.692
3 mos postop	34.7 ± 18.1	26.0 ± 13.2	0.169
2 year follow-up	36.8 ± 18.6	28.9 ± 14.7	0.240
Postop change	-18.2 ± 15.5	-22.7 ± 15.9	0.481
TK (°)			
Preop	44.0 ± 17.6	39.8 ± 16.1	0.441
Immed. postop	32.1 ± 14.4	26.9 ± 13.4	0.355
2 year follow-up	34.2 ± 15.7	29.6 ± 13.2	0.419
Postop change	-5.7 ± 12.7	-10.3 ± 13.5	0.393
TLK (°)			
Preop	30.6 ± 14.7	26.9 ± 19.3	0.599
3 mos postop	13.8 ± 9.4	1.1 ± 19.4	0.057
2 year follow-up	13.4 ± 11.9	-0.1 ± 16.6	0.031*
Postop change	-17.2 ± 17.1	-27.0 ± 12.8	0.107
LL (°)			
Preop	18.3 ± 12.6	4.9 ± 29.3	0.039*
3 mos postop	31.3 ± 8.9	31.4 ± 13.8	0.877
2 year follow-up	28.6 ± 9.3	24.9 ± 27.6	0.621
Postop change	13.1 ± 15.5	26.5 ± 21.6	0.047*
PT (°)			
Preop	28.3 ± 12.4	32.4 ± 12.6	0.421
3 mos postop	21.7 ± 9.2	25.0 ± 10.5	0.418
2 year follow-up	24.7 ± 10.4	26.3 ± 11.3	0.711
Postop change	-3.6 ± 3.5	-6.0 ± 4.7	0.159
SS (°)			
Preop	12.6 ± 6.4	15.7 ± 8.2	0.311
3 mos postop	18.1 ± 7.6	21.4 ± 7.9	0.287
2 year follow-up	16.4 ± 6.9	21.2 ± 7.9	0.124
Postop change	3.8 ± 4.2	5.5 ± 6.1	0.438
PI (°)			
Preop	40.9 ± 17.3	47.2 ± 17.5	0.375
3 mos postop	40.0 ± 17.0	46.7 ± 17.3	0.335
2 year follow-up	41.2 ± 17.3	47.1 ± 17.5	0.402
Postop change	0.3 ± 1.5	-0.0 ± 1.7	0.615

*C7SVA *C7 sagittal vertical axis, *GK *global kyphosis, *TK *thoracic kyphosis, *TLK *thoracolumbar kyphosis, *LL *lumbar lordosis, *PT *pelvic tilt, *SS *sacral slope, *PI *pelvic incidence

* Statistically significant difference between two groups at the indicated time point (*p* < 0.05).

**Table 3:** Comparison of Patients Reported Outcome Measures in the IS Group and VB Group VAS: visual analog scale, ODI: Oswestry disability index

	IS Group (*n* = 17)	VB Group (*n* = 24)	*p*-value
VAS			
Preop	6.5 ± 1.1	6.7 ± 1.2	0.431
3 mos postop	4.1 ± 0.8	3.9 ± 0.7	0.261
2 year follow-up	2.8 ± 0.8	2.8 ± 2.5	0.649
Final change	-3.6 ± 1.2	-4.1 ± 1.1	0.142
ODI			
Preop	62.1 ± 7.9	60.9 ± 5.1	0.725
3 mos postop	31.3 ± 3.7	30.1 ± 3.4	0.241
2 year follow-up	18.6 ± 1.8	19.1 ± 2.6	0.306
Final change	-41.0 ± 5.9	-43.0 ± 5.7	0.254

*VAS *visual analog scale, *ODI *Oswestry disability index

**Table 4:** Postoperative Complications in the IS Group and VB Group

	IS Group (*n* = 17)	VB Group (*n* = 24)	*p*-value
Complication (n, %)	2 (11.8%)	9 (37.5%)	0.085
Dural tear	2 (11.8%)	5 (20.8%)	0.679
Deep infection	1 (5.9%)	2 (8.3%)	1.0
Pneumothorax	0	1 (4.2%)	1.0
Neurologic deficit	0	1 (4.2%)	1.0

†One IS-group patient sustained both a dural tear and a deep infection; complications are reported by event count

### Propensity score-matched analysis

Seventeen matched pairs (*n* = 17 per group) were identified via propensity score matching. Post-matching covariate balance is presented in Table 1. After matching, baseline demographic variables and most radiographic parameters achieved acceptable balance (SMD < 0.1). However, C7SVA remained imbalanced (SMD = 0.680), reflecting the intrinsic morphological distinction between VB- and IS-type fractures rather than residual matching bias. The VB group continued to demonstrate significantly greater postoperative sagittal correction compared to the IS group (ΔC7SVA: 126.4 ± 29.7 mm (VB) vs. 72.5 ± 61.8 mm (IS); *p* = 0.004, ΔLL: 22.0 ± 11.3° (VB) vs. 13.1 ± 15.5° (IS); *p* = 0.066). Pain (VAS) and disability (ODI) improvements remained statistically similar between groups in the matched cohort. Complications occurred in 6 VB-type patients (35.3%) versus 2 IS-type patients (11.8%); this difference did not reach statistical significance *(p* = 0.225), and a type II error due to limited sample size cannot be excluded.

### Predictive modeling

#### Multivariable logistic regression

Multivariable logistic regression was performed to identify independent preoperative predictors of PSO selection. Among the three candidate variables, C7SVA was the only statistically significant predictor (β = 0.013; OR = 1.013; 95% CI: 1.001–1.026; *p* = 0.039), indicating that each 1-mm increase in preoperative C7SVA was associated with a 1.3% increase in the odds of undergoing PSO. Although the overall likelihood ratio test for the multivariable model did not reach statistical significance (LLR *p* = 0.125), likely reflecting the limited sample size and modest model fit, C7SVA remained individually significant as an independent predictor. LL (OR = 1.007; 95% CI: 0.977–1.038; *p* = 0.648) and BMD (OR = 0.925; 95% CI: 0.627–1.363; *p* = 0.692) did not reach statistical significance, likely reflecting limited statistical power in this cohort. VIF values were 1.25 for C7SVA and 1.09 for LL, and 1.16 for BMD, all well below the accepted threshold of 5.0, confirming acceptable multicollinearity. Full regression results are presented in Table 5. The logistic regression model demonstrated modest discriminative ability (AUC = 0.694), which may be attributable to the limited sample size and model parsimony.

**Table 5:** Uni- and multivariable logistic regression for predictors of PSO vs. non-PSO surgical strategy

Variable	OR	95% CI	*p*-value	OR	95% CI	*p*-value
Patient characteristics						
Age (years)	0.981	0.929–1.037	0.508	—	—	—
Sex (Male)	1.185	0.230–6.120	0.839	—	—	—
BMD (T-score)	0.916	0.639–1.315	0.635	0.925	0.627–1.363	0.692
Fracture type (VB vs. IS)	48.000	5.205–442.642	**< 0.001**	—	—	—
Pre-operative radiographic parameters						
C7SVA (mm)	1.014	1.001–1.027	**0.037**	1.013	1.001–1.026	**0.039**
LL (°)	1.009	0.981–1.039	0.527	1.007	0.977–1.038	0.648
GK (°)	0.987	0.957–1.019	0.428	—	—	—
TLK (°)	0.988	0.953–1.024	0.502	—	—	—
PI (°)	0.984	0.941–1.030	0.493	—	—	—
PT (°)	0.994	0.935–1.057	0.844	—	—	—
SS (°)	0.976	0.900–1.057	0.546	—	—	—
Model performance (Multivariable)						
AUC = 0.694 | Pseudo R² = 0.101 | LLR *p* = 0.125 | AIC = 58.9 | *n* = 41						
* VIF: C7SVA = 1.252 | LL = 1.089 | BMD = 1.161*						

Outcome: pedicle subtraction osteotomy (PSO) vs. non-PSO (*n* = 41)

*OR* odds ratio, *CI *confidence interval, *BMD *bone mineral density, *C7SVA *C7 sagittal vertical axis, *LL *lumbar lordosis, *VIF *variance inflation factor. *AUC *area under the receiver operating characteristic curve, Pseudo R², McFadden’s pseudo R-squared; LLR p, likelihood ratio test p-value; *AIC *Akaike information criterion, *VIF *variance inflation factor

Bold p-values indicate statistical significance (*p* < 0.05)

† Multivariable model variables were selected a priori based on clinical relevance (C7SVA, LL) and bone quality (BMD); non-significant predictors were retained for completeness. VIF < 5 indicates no multicollinearity concern

### Decision tree analysis

**Fig. 1:**
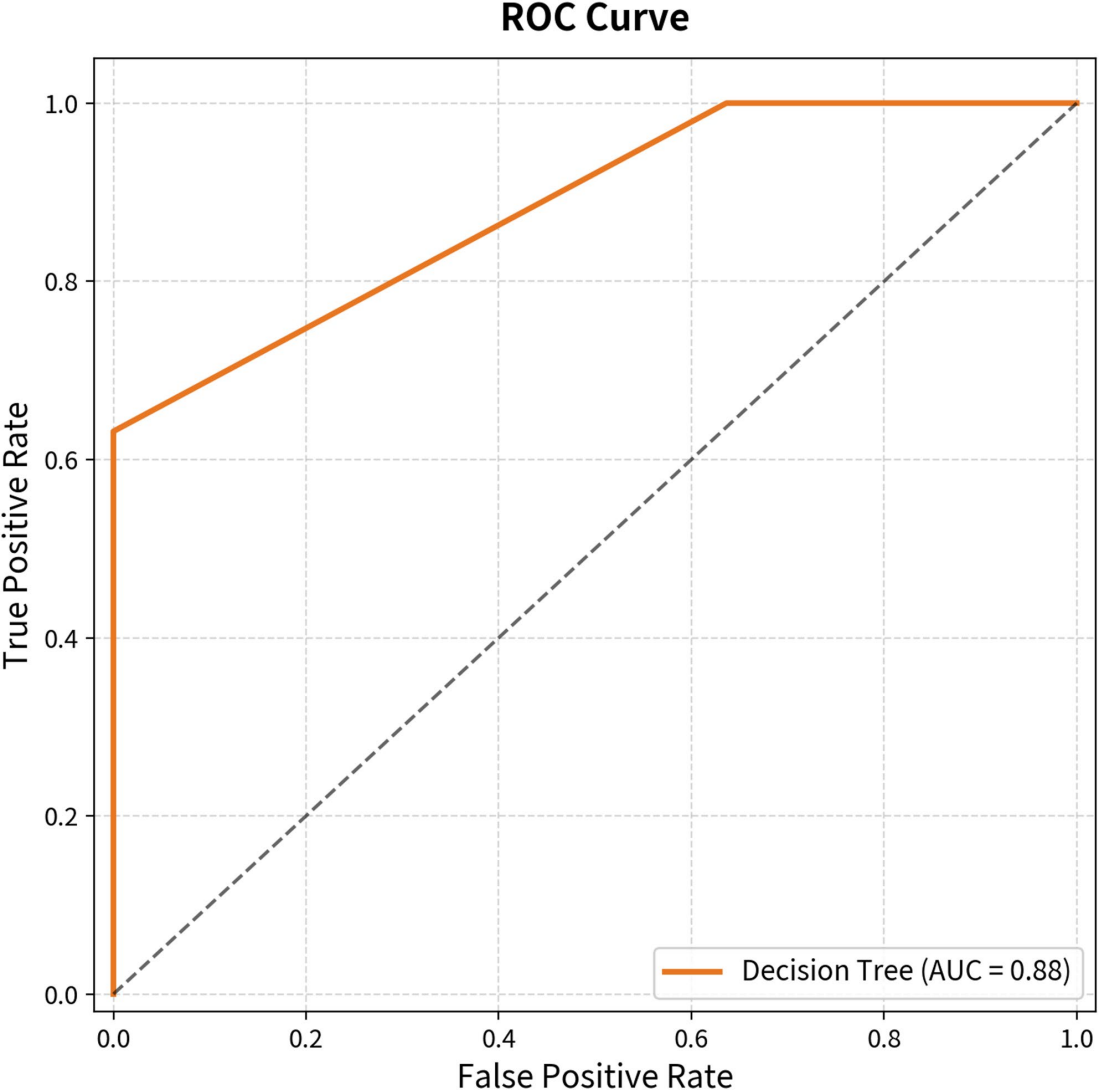
Receiver operating characteristic (ROC) curve showing performance of the decision tree model for predicting surgical strategy (PSO vs. non-PSO). The area under the curve (AUC) was 0.88 (95% CI: 0.85–0.99). Leave-one-out cross-validation (LOOCV) AUC = 0.77, indicating stable model performance despite the limited sample size

**Fig. 2:**
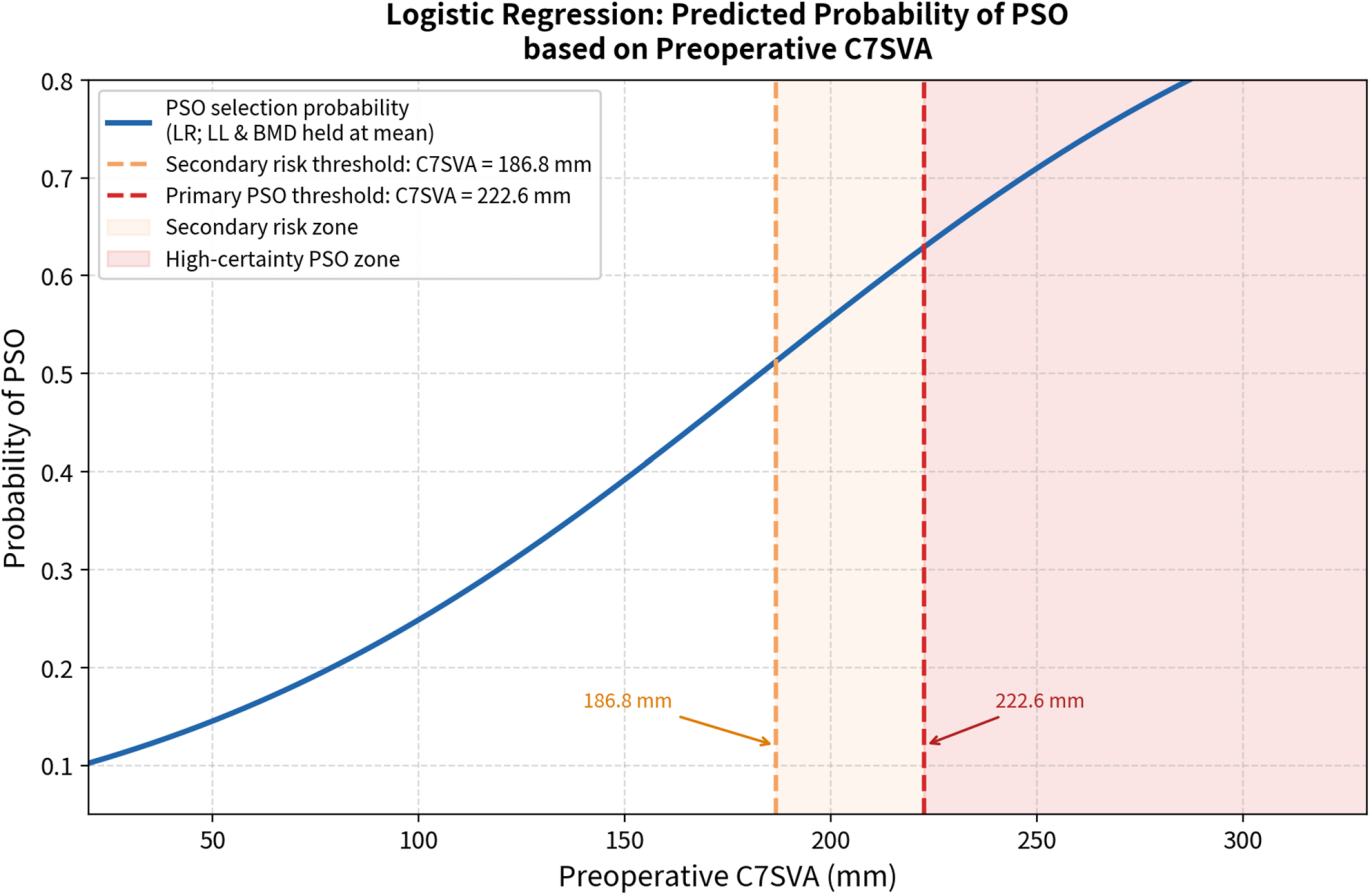
Logistic regression curve illustrating the predicted probability of selecting PSO based on preoperative C7SVA (lumbar lordosis and BMD held at mean values). The orange dashed line indicates the secondary risk threshold (C7SVA = 186.8 mm) and the red dashed line indicates the primary PSO threshold (C7SVA = 222.6 mm) derived from decision tree analysis

**Fig. 3:**
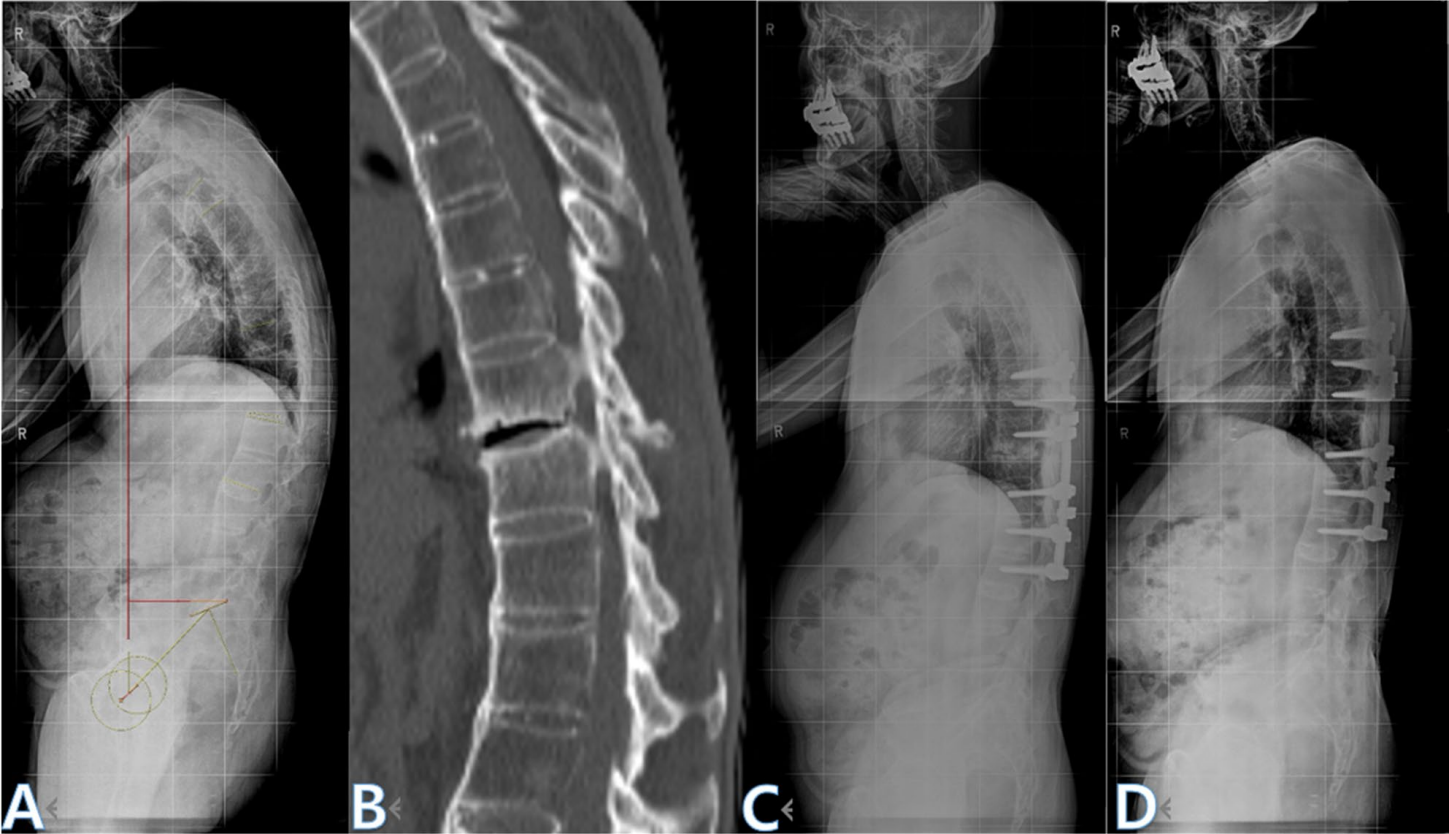
Representative case of an intervertebral space (IS-type) fracture treated with PSF. **A** Preoperative lateral radiograph showing kyphotic deformity. **B **CT scan demonstrating a disc space fracture at T11–12. **C **Radiograph at 3 months postoperative showing realignment after PSF. **D **Final follow-up (2 years) radiograph showing union and maintained correction

**Fig. 4:**
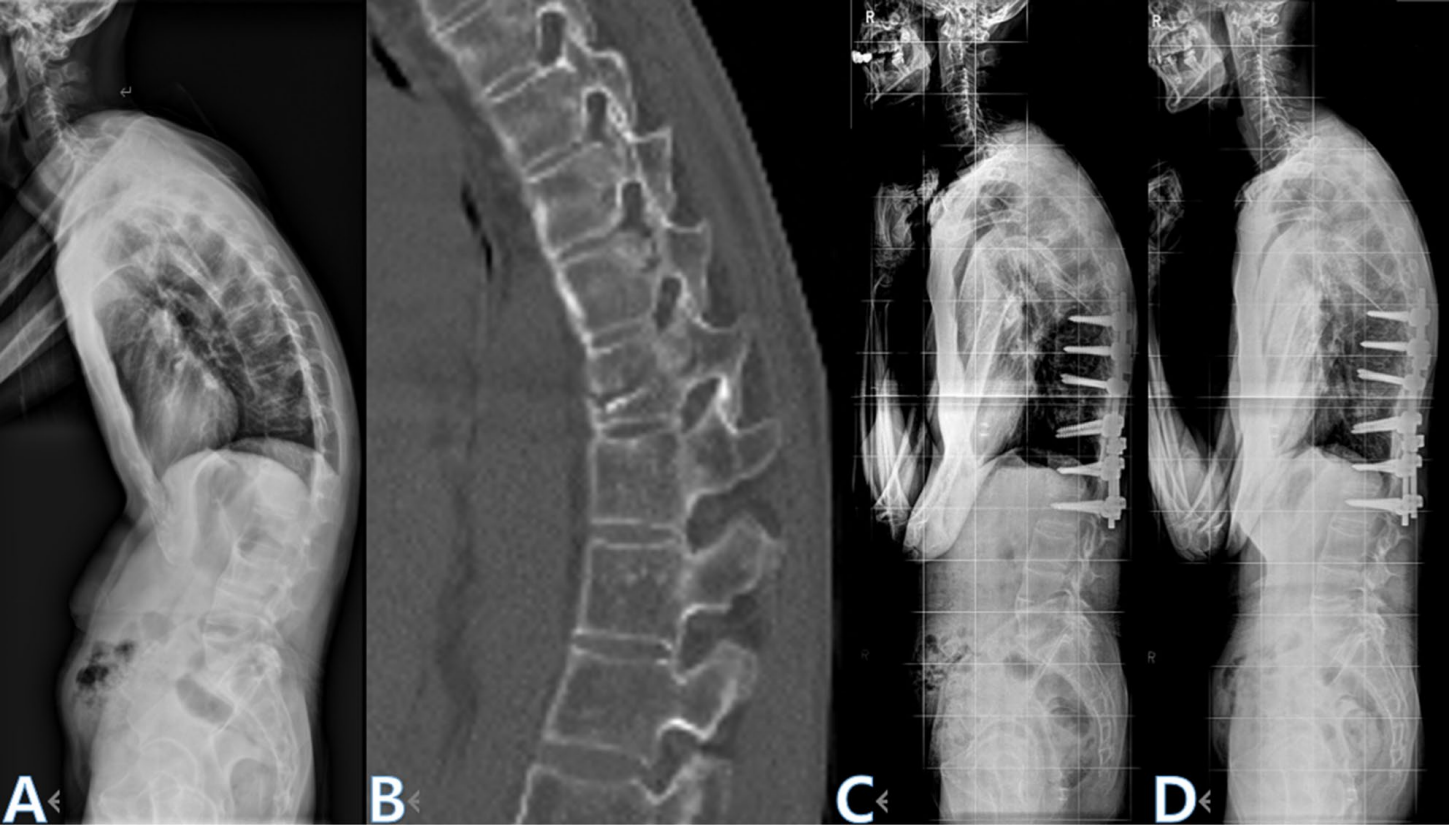
Representative case of a vertebral body (VB-type) fracture treated with PSO. **A **Preoperative lateral radiograph demonstrating sagittal malalignment. **B **CT scan showing a 3-column vertebral body fracture at T11. **C **3-month postoperative radiograph after PSO and fusion from T8–L2. **D **Final follow-up radiograph showing maintained correction and fusion

A CART decision tree model (Gini impurity; maximum depth = 3) was constructed to generate a clinically applicable classification algorithm. The model achieved a training accuracy of 82.9% and AUC = 0.883 (95% CI: 0.851–0.992). Leave-one-out cross-validation (LOOCV) confirmed model stability, yielding a consistent accuracy of 82.9% and AUC = 0.766 across all 41 iterations. C7SVA > 222.6 mm served as the primary splitting criterion in the final model, uniformly classifying patients as requiring PSO. Among patients with C7SVA ≤ 222.6 mm, a secondary split at C7SVA > 186.8 mm combined with BMD ≤ − 2.25 further stratified PSO versus non-PSO cases (Figs. 1, 2, 3, 4). These thresholds are consistent with the logistic regression finding that C7SVA was the dominant radiographic determinant of surgical strategy, supporting a data-driven, stepwise decision framework for individualized surgical planning.

## Discussion

This study aimed to evaluate the safety and outcomes of PSO-based deformity correction compared to non-PSO strategies in AS patients with thoracolumbar fractures, and to identify preoperative radiographic thresholds that could guide surgical decision-making. Using a propensity score-matched cohort and predictive modeling, we demonstrated that fracture morphology — specifically VB-type versus IS-type classification — is strongly associated with preoperative sagittal imbalance, the selection of surgical strategy, and complication risk.

### Interpretation of surgical strategy by fracture type

The classification of fractures into VB-type and IS-type provided a clinically relevant distinction with strong implications for surgical planning. VB-type fractures typically involve three-column disruption through the vertebral body and result in severe kyphotic collapse and mechanical instability [1, 2, 4]. This was reflected in our cohort by significantly elevated preoperative C7SVA (VB: 201.1 ± 60.7 mm vs. IS: 139.9 ± 65.6 mm; *p* < 0.001) and, importantly, significantly reduced lumbar lordosis (LL; VB: 4.9 ± 29.3° vs. IS: 18.3 ± 12.6°; *p* = 0.039) at baseline. Although PSM reduced confounding from most demographic and radiographic variables, a residual difference in C7SVA persisted after matching (SMD = 0.680), reflecting the fundamental morphological distinction between fracture types that could not be fully eliminated by matching alone. These patients more commonly underwent PSO, which offers robust sagittal realignment but is technically demanding and more invasive.

In contrast, IS-type fractures localized to the intervertebral disc space generally preserved partial columnar integrity and were associated with milder sagittal malalignment. Notably, IS-type patients maintained a mean preoperative LL of 18.3 ± 12.6°, indicating relatively preserved lumbar curvature despite the fracture. This partial preservation of lordosis — combined with lower C7SVA — meant that posterior stabilization alone was sufficient to prevent further deformity progression, without the need for active osteotomy-based realignment. Patients in this group more often received PSF, either in situ or augmented with structural bone graft. This less invasive approach achieved adequate stabilization without requiring correction of the deformity in most cases.

These findings are supported by prior studies indicating that VB-type fractures in AS represent structurally unstable lesions prone to progressive collapse and require more aggressive surgical correction [7, 9, 10, 13]. The intervertebral space, by contrast, may retain segmental stability and tolerate posterior fixation alone when the sagittal balance is relatively preserved [14, 15]. Importantly, the choice of surgical strategy in this study was not based on a predefined protocol but rather followed the general principles used in thoracolumbar fractures [2, 15]. 

### Radiographic correction vs. functional outcome

While the VB group demonstrated significantly greater correction in both C7SVA (ΔC7SVA: −132.7 ± 48.9 mm vs. −72.5 ± 61.8 mm; *p* < 0.001) and LL (ΔLL: +26.5 ± 21.6° vs. +13.1 ± 15.5°; *p* = 0.047), the magnitude of deformity correction did not translate into superior clinical outcomes at the follow-up interval studied. The substantial LL restoration achieved via PSO (mean gain of 26.5°) brought VB-type patients from a near-flat lumbar profile (4.9°) to a functionally adequate lordosis (31.4° at 3 months), underscoring PSO’s unique capacity to address both the translational (C7SVA) and angular (LL) components of sagittal deformity in this population. Visual Analog Scale (VAS) and Oswestry Disability Index (ODI) scores improved comparably in both groups. This indicates that although sagittal realignment supports biomechanical restoration and long-term function, it does not necessarily yield proportional short-term symptom relief—especially when neural elements are not severely compromised preoperatively.

One possible explanation is that deformity correction addresses global biomechanics but may not directly impact localized pain generators such as facet joint strain or muscular compensation. Additionally, the more extensive tissue disruption required for PSO could transiently negate some of the early functional gains. Nevertheless, in patients with marked sagittal imbalance or loss of horizontal gaze, correction may prevent future compensatory mechanisms, improve energy efficiency, and reduce fall risk [6, 14]. 

### Surgical risk and complication profile

We observed a trend toward higher complication rates in the VB group, particularly among patients who underwent PSO (37.5% vs. 11.8%, *p* = 0.085). Although this difference did not reach statistical significance, it is likely attributable to limited statistical power (Type II error) rather than a true absence of clinical difference, given the small sample size. The observed complications -including dural tears, deep infections, transient neurological deficits, and pneumothorax- are consistent with previously published reports on complex osteotomy procedures in rigid, osteoporotic spines [16, 17]. The risk- benefit profile of PSO must therefore be carefully individualized, particularly in elderly patients with significant medical comorbidities. For patients with less severe sagittal deformity, PSF with or without structural bone grafting may offer comparable symptom relief with a lower procedural risk.

### Predictive modeling and algorithmic decision-making

A major strength of this study lies in the development of a predictive algorithm to guide preoperative surgical decision-making. The decision tree model — constructed using the CART algorithm with Gini impurity and a maximum depth of 3 — achieved a training accuracy of 82.9% and AUC = 0.883 (95% CI: 0.851–0.992). Leave-one-out cross-validation confirmed model stability, with a consistent LOOCV accuracy of 82.9% and LOOCV AUC of 0.766, suggesting that the model generalizes reasonably well despite the small sample size.

The tree identified two clinically meaningful C7SVA thresholds. Patients with C7SVA > 222.6 mm were uniformly classified as requiring PSO, representing a high-certainty indication for aggressive deformity correction. Among patients with C7SVA ≤ 222.6 mm, a secondary split at C7SVA > 186.8 mm further stratified the cohort, with BMD ≤ − 2.25 serving as an additional determinant in the lower C7SVA range — highlighting the compounding influence of osteoporosis on surgical decision-making in this population.

Complementing these findings, multivariable logistic regression identified C7SVA as the only statistically significant independent predictor of PSO selection (OR = 1.013; 95% CI: 1.001–1.026; *p* = 0.039), consistent with the decision tree’s primary splitting criterion. LL and BMD did not reach statistical significance in the multivariable regression model (LL: OR = 1.007; *p* = 0.648), despite LL showing a statistically significant between-group difference at baseline (*p* = 0.039). This apparent discrepancy likely reflects the collinearity between LL and C7SVA — two mechanically linked parameters — and the limited statistical power of this cohort. The non-significance of LL in the regression model should therefore not be interpreted as a lack of clinical relevance; rather, LL reduction serves as a mechanistic correlate of the sagittal imbalance captured by C7SVA, and its preoperative assessment remains an important component of comprehensive sagittal profiling. The convergence of two independent analytical methods on C7SVA as the dominant predictor strengthens confidence in this threshold as a clinically meaningful decision point.

These findings suggest that a straightforward preoperative sagittal radiographic assessment, centered on C7SVA measurement, can serve as a practical foundation for algorithmic surgical planning in AS-related thoracolumbar fractures. From a clinical standpoint, the finding that VB-type fractures are associated with both severe C7SVA elevation and significant LL reduction has direct therapeutic implications. When preoperative C7SVA exceeds 222.6 mm or LL falls below approximately 5°, posterior fusion alone is unlikely to restore adequate sagittal alignment, and PSO should be planned preoperatively rather than decided intraoperatively. Conversely, IS-type patients with preserved LL (> 15°) and moderate C7SVA (< 187 mm) can typically be managed with PSF, sparing them the additional morbidity of osteotomy. Integrating both C7SVA and LL into preoperative decision frameworks — rather than relying on C7SVA alone — may therefore improve patient stratification and surgical planning in this challenging population.

### Comparison with previous literature

Few prior studies have applied radiographic thresholds to inform surgical strategy in AS-related spinal fractures. Most existing literature consists of case series or technique-focused reports. For example, Qian et al. and Guo et al. describe successful sagittal correction via PSO through pseudarthrosis in AS, but do not compare different fracture types [7, 9]. Our study fills this gap by comparing two clinically distinct morphologies and establishing a reproducible selection algorithm.

Moreover, while prior studies have emphasized surgical technique or hardware choice, our focus on preoperative sagittal alignment provides an upstream approach—allowing for individualized planning before the incision is made. This has potential to not only optimize outcomes but also reduce unnecessary surgical morbidity in this high-risk population.

### Study limitations

This study has several inherent limitations that should be considered when interpreting the findings. First, the retrospective single-center design introduces the potential for selection and observer bias, although propensity score matching was employed to mitigate baseline confounding. Second, the small sample size (*n* = 41) limits statistical power, particularly for detecting differences in complication rates (type II error cannot be excluded), and reduces the precision of model performance estimates — as reflected by the wide bootstrapped confidence interval for the decision tree AUC (95% CI: 0.851–0.992). Third, the predictive models were developed and evaluated on the same dataset without an independent external validation cohort, which limits the generalizability of the proposed thresholds. Leave-one-out cross-validation was applied to partially address this concern, yielding a LOOCV AUC of 0.766, which represents a more conservative and realistic estimate of model performance. Fourth, the surgical strategy was determined by individual surgeon judgment rather than a standardized protocol, which may introduce unmeasured decision-making variability. Future multicenter studies with larger cohorts are needed to prospectively validate the proposed C7SVA-based decision algorithm and assess its real-world clinical utility.

## Conclusion

In AS patients with thoracolumbar fractures, VB-type injuries with marked sagittal imbalance more frequently require PSO-based deformity correction, whereas IS-type injuries can generally be managed with PSF alone. A C7SVA > 222.6 mm represented a high likelihood of PSO, whereas C7SVA > 186.8 mm combined with BMD ≤ − 2.25 identified a secondary risk group. These data-driven thresholds offer a practical framework for individualized surgical planning, though prospective external validation in larger cohorts is warranted.

## Supplementary Information

Supplementary Material 1.

## Data Availability

The datasets generated and/or analyzed during the current study are not publicly available due to the inclusion of sensitive human clinical data but are available from the corresponding author on reasonable request.
