# Supplementary material for: Fracture morphology–driven surgical strategy in ankylosing spondylitis: when does sagittal imbalance mandate pedicle subtraction osteotomy?
Source: BMC Musculoskelet Disord. 2026 Apr 11;27:435. doi: 10.1186/s12891-026-09806-w (PMC13191913; doi:10.1186/s12891-026-09806-w)
Supplement: Supplementary file 1 — Supplementary Material 1. [file 12891_2026_9806_MOESM1_ESM.zip › Table.docx]

Table 1. Demographic Characteristics by Fracture Location

*BMI:* body mass index, *BMD:* bone mineral density, PSF: posterior spinal fusion, PSO: pedicle subtraction osteotomy, BG: bone graft

| Table 2. Comparison of Radiologic Parameters in the IS Group and VB Group | | | |
| --- | --- | --- | --- |
|  | IS Group (n=17) | VB Group (n=24) | *p*-v*alue* |
| **C7SVA (mm)** | | | |
| Preop | 139.9 ± 65.6 | 201.1 ± 60.7 | ***< 0.001**** |
| 3 mos postop | 67.4 ± 39.2 | 68.4 ± 20.9 | *0.718* |
| 2yr follow-up | 70.8 ± 29.3 | 76.3 ± 24.9 | *0.324* |
| Postop change | -72.5 ± 61.8 | -132.7 ± 48.9 | ***< 0.001**** |
| **GK (°)** | | | |
| Preop | 55.0 ± 23.5 | 51.6 ± 19.8 | *0.692* |
| 3 mos postop | 34.7 ± 18.1 | 26.0 ± 13.2 | *0.169* |
| 2 yr follow-up | 36.8 ± 18.6 | 28.9 ± 14.7 | *0.240* |
| Postop change | -18.2 ± 15.5 | -22.7 ± 15.9 | *0.481* |
| **TK (°)** | | | |
| Preop | 44.0 ± 17.6 | 39.8 ± 16.1 | *0.985* |
| Immed. postop | 32.1 ± 14.4 | 26.9 ± 13.4 | *0.355* |
| 2 yr follow-up | 34.2 ± 15.7 | 29.6 ± 13.2 | *0.419* |
| Postop change | -5.7 ± 12.7 | -10.3 ± 13.5 | *0.393* |
| **TLK (°)** | | | |
| Preop | 30.6 ± 14.7 | 26.9 ± 19.3 | *0.599* |
| 3 mos postop | 13.8 ± 9.4 | 1.1 ± 19.4 | *0.057* |
| 2 yr follow-up | 13.4 ± 11.9 | -0.1 ± 16.6 | ***0.031**** |
| Postop change | -17.2 ± 17.1 | -27.0 ± 12.8 | *0.107* |
| **LL (°)** | | | |
| Preop | 18.3 ± 12.6 | 4.9 ± 29.3 | ***0.039**** |
| 3 mos postop | 31.3 ± 8.9 | 31.4 ± 13.8 | 0.877 |
| 2 yr follow-up | 28.6 ± 9.3 | 24.9 ± 27.6 | 0.621 |
| Postop change | 13.1 ± 15.5 | 26.5 ± 21.6 | ***0.047**** |
| **PT (°)** | | | |
| Preop | 28.3 ± 12.4 | 32.4 ± 12.6 | *0.421* |
| 3 mos postop | 21.7 ± 9.2 | 25.0 ± 10.5 | *0.418* |
| 2 yr follow-up | 24.7 ± 10.4 | 26.3 ± 11.3 | *0.711* |
| Postop change | -3.6 ± 3.5 | -6.0 ± 4.7 | *0.159* |
| **SS (°)** | | | |
| Preop | 12.6 ± 6.4 | 15.7 ± 8.2 | *0.311* |
| 3 mos postop | 18.1 ± 7.6 | 21.4 ± 7.9 | *0.287* |
| 2 yr follow-up | 16.4 ± 6.9 | 21.2 ± 7.9 | *0.124* |
| Postop change | 3.8 ± 4.2 | 5.5 ± 6.1 | *0.438* |
| **PI (°)** | | | |
| Preop | 40.9 ± 17.3 | 47.2 ± 17.5 | *0.375* |
| 3 mos postop | 40.0 ± 17.0 | 46.7 ± 17.3 | *0.335* |
| 2 yr follow-up | 41.2 ± 17.3 | 47.1 ± 17.5 | *0.402* |
| Postop change | 0.3 ± 1.5 | -0.0 ± 1.7 | *0.615* |
| C7SVA: C7 sagittal vertical axis; TK: thoracic kyphosis, TLK: thoracolumbar kyphosis; LL: lumbar lordosis; PT: pelvic tilt; SS: sacral slope; PI: pelvic incidence  * A statistically significant difference in parameters between preoperative and postoperative within the group (*p*<0.05) | | | |

Table 3. Comparison of Patients Reported Outcome Measures in the IS Group and VB Group

|  | IS Group (n=17) | VB Group (n=24) | *p*-v*alue* |
| --- | --- | --- | --- |
| VAS | | | |
| Preop | 6.5±1.1 | 6.7±1.2 | *0.431* |
| 3 mos postop | 4.1±0.8 | 3.9±0.7 | *0.261* |
| 2yr follow-up | 2.8±0.8 | 2.8±2.5 | *0.649* |
| Final change | -3.6 ± 1.2 | -4.1 ± 1.1 | *0.142* |
| ODI |  |  |  |
| Preop | 62.1±7.9 | 60.9±5.1 | *0.725* |
| 3 mos postop | 31.3±3.7 | 30.1±3.4 | *0.241* |
| 2yr follow-up | 18.6±1.8 | 19.1±2.6 | *0.306* |
| Final change | -41.0±5.9 | -43.0±5.7 | *0.254* |

VAS: visual analog scale, ODI: Oswestry disability index

* A statistically significant difference in parameters between preoperative and postoperative within the group (*p*<0.05)

| Table 4. Postoperative Complications in the IS Group and VB Group | | |  |
| --- | --- | --- | --- |
|  | IS Group (n=17) | VB Group (n=24) | *p-value* |
| Complication (n, %) | 2 (11.8%) | 9 (37.5%) | 0.085 |
| Dural tear | 2 (11.8%) | 5 (20.8%) | 0.679 |
| Deep infection | 1 (5.9%) | 2 (8.3%) | 1.0 |
| Pneumothorax | 0 | 1 (4.2%) | 1.0 |
| Neurologic deficit | 0 | 1 (4.2%) | 1.0 |

* A statistically significant difference in parameters between IS and VB group (*p*<0.05)
