# Supplementary figures and images for: Fracture morphology–driven surgical strategy in ankylosing spondylitis: when does sagittal imbalance mandate pedicle subtraction osteotomy?
Source: BMC Musculoskelet Disord. 2026 Apr 11;27:435. doi: 10.1186/s12891-026-09806-w (PMC13191913; doi:10.1186/s12891-026-09806-w)

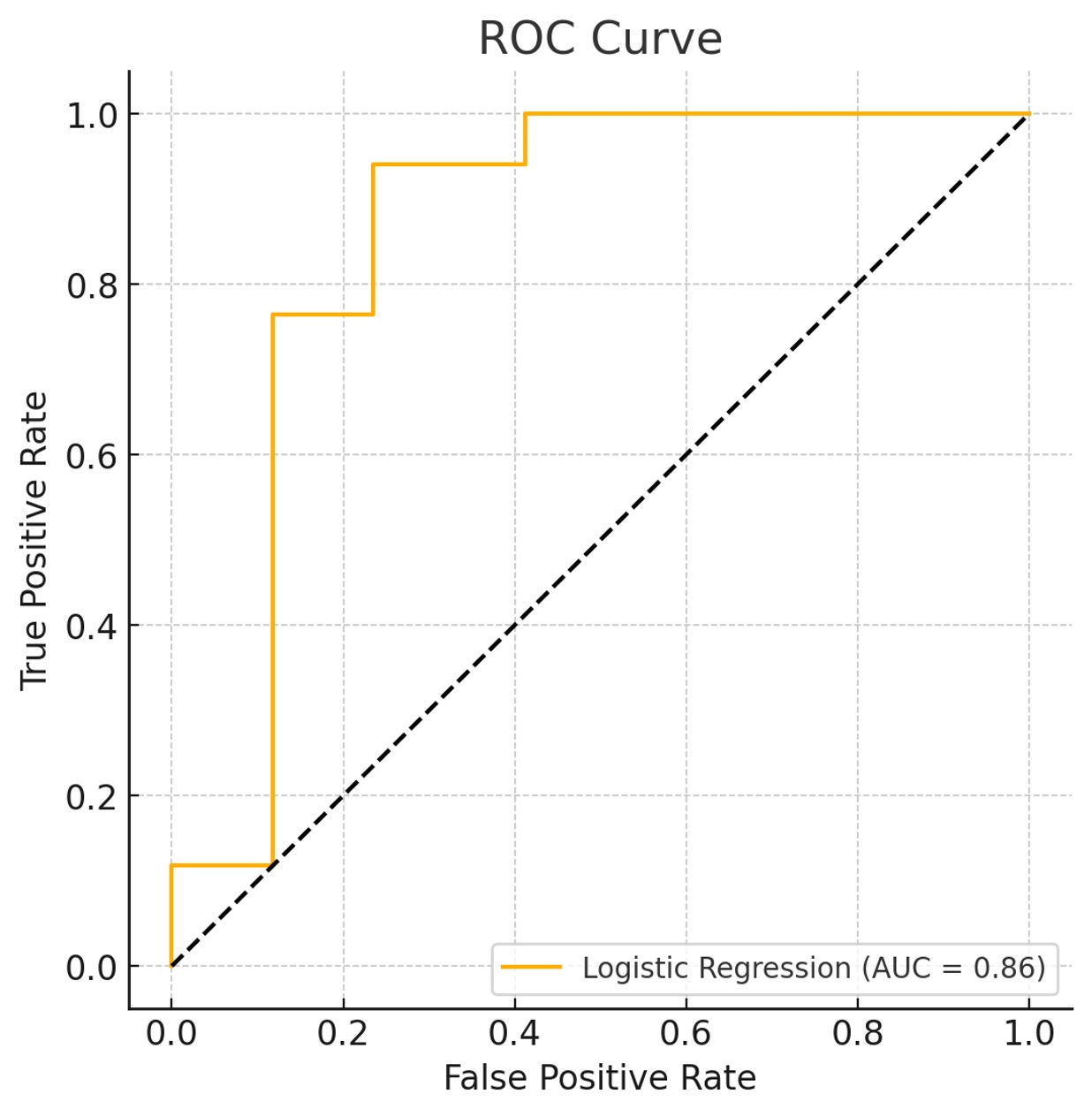

Supplement: Supplementary file 1 — Supplementary Material 1. [file 12891_2026_9806_MOESM1_ESM.zip › Figure 1.jpg]

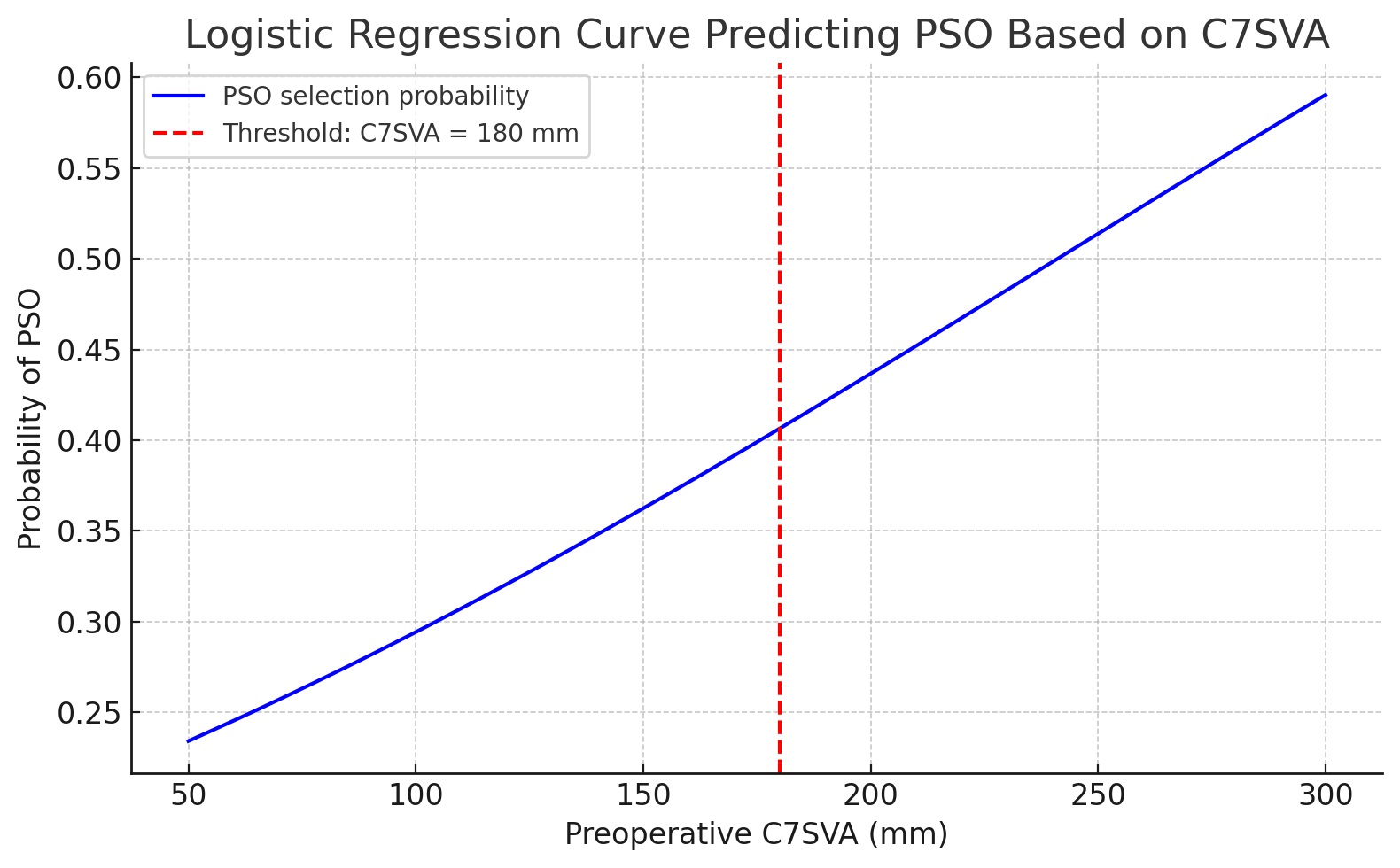

Supplement: Supplementary file 1 — Supplementary Material 1. [file 12891_2026_9806_MOESM1_ESM.zip › Figure 2.jpg]

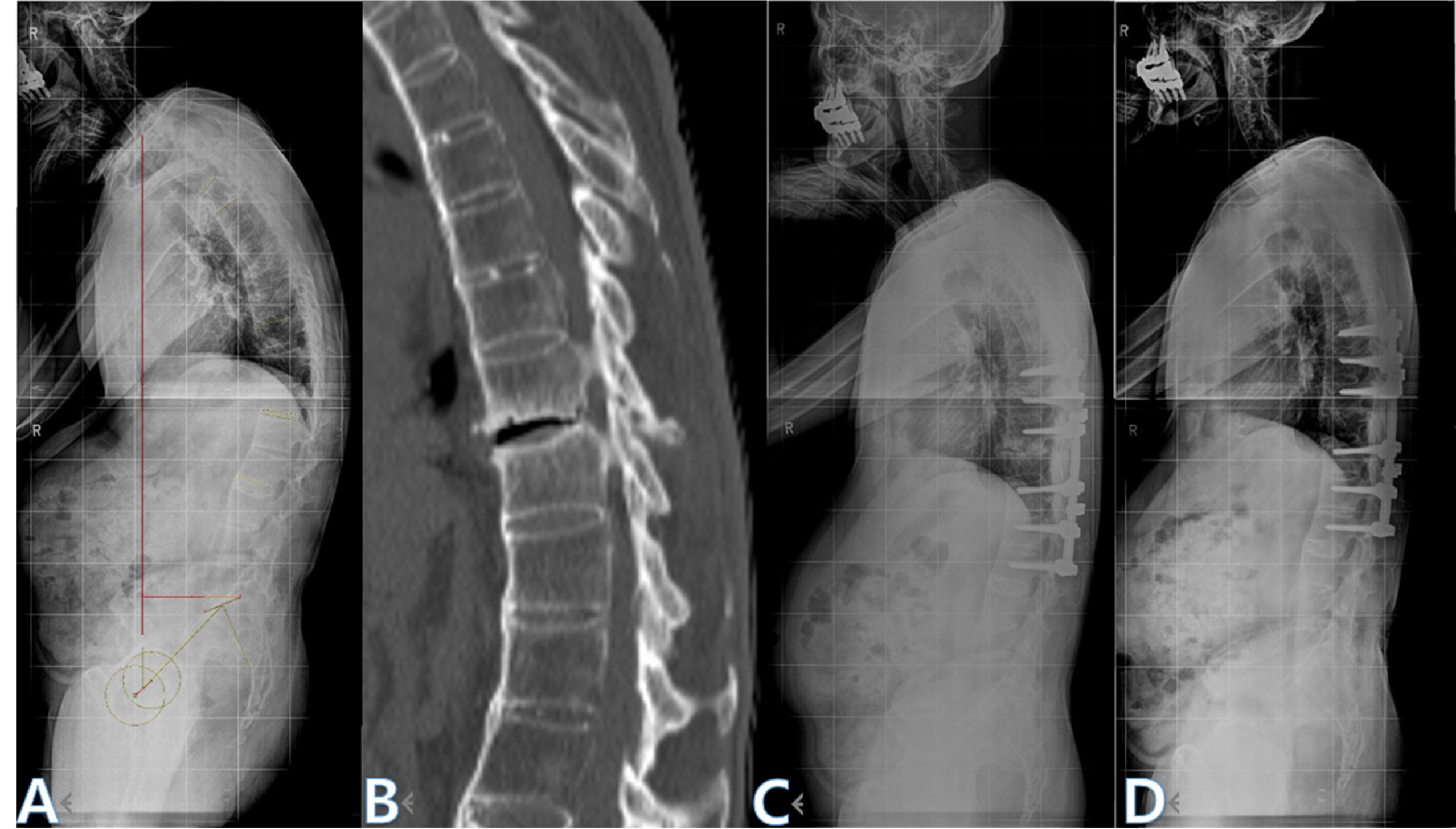

Supplement: Supplementary file 1 — Supplementary Material 1. [file 12891_2026_9806_MOESM1_ESM.zip › Figure 3.JPG]

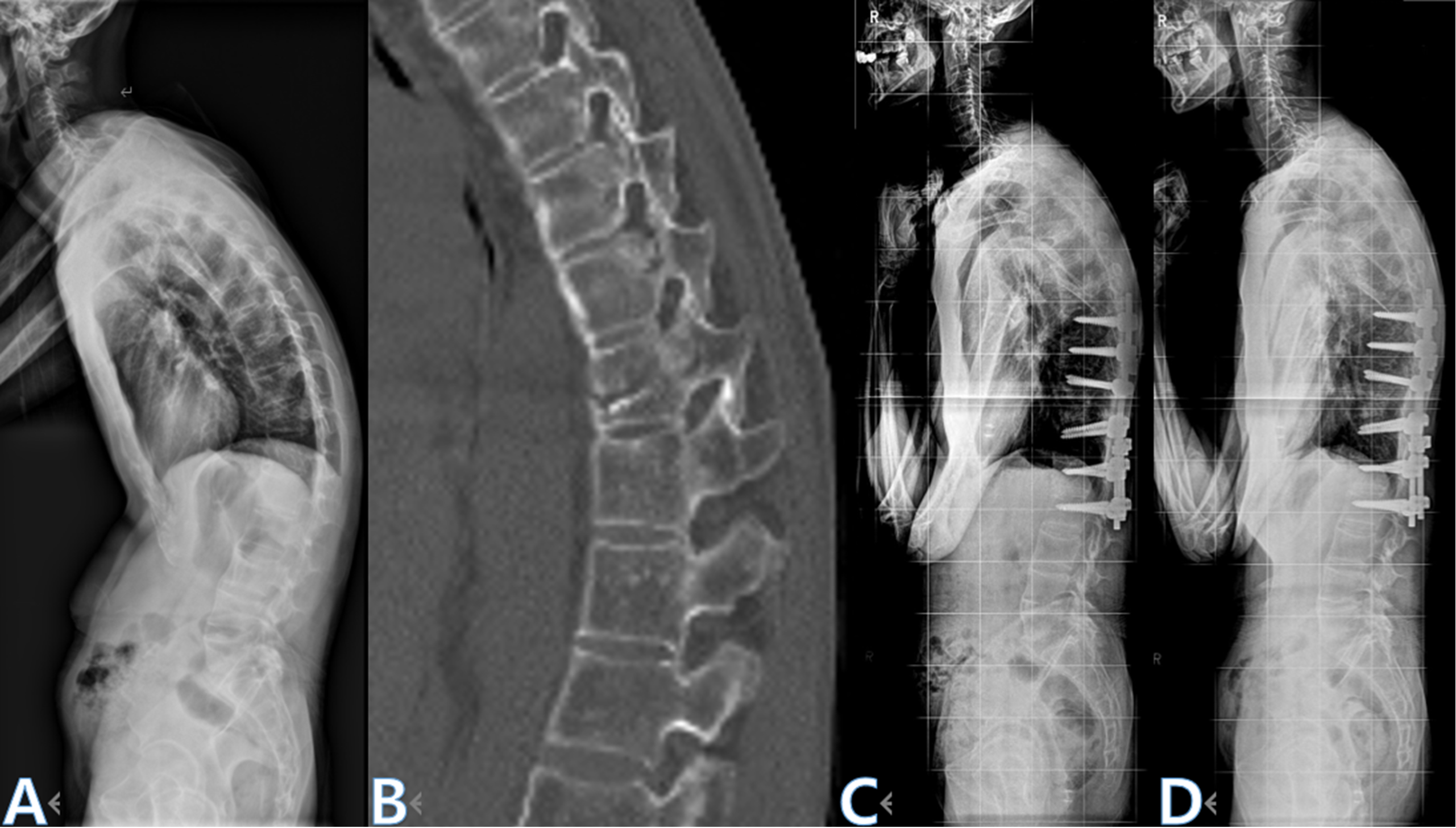

Supplement: Supplementary file 1 — Supplementary Material 1. [file 12891_2026_9806_MOESM1_ESM.zip › Figure 4.JPG]
